# Dataset of five years of *in situ* and satellite derived chlorophyll *a* concentrations and its spatiotemporal variability in the Rotorua Te Arawa Lakes, New Zealand

**DOI:** 10.1016/j.dib.2021.107759

**Published:** 2021-12-23

**Authors:** Eike M Schütt, Moritz K Lehmann, Martin Hieronymi, James Dare, Hajo Krasemann, Darryn Hitchcock, Amy Platt, Klay Amai, Tasman McKelvey

**Affiliations:** a Institute of Coastal Ocean Dynamics, Helmholtz-Zentrum Hereon, Max-Planck-Straße 1, Geesthacht 21502, Germany; b Xerra Earth Observation Institute, 17a Brandon Street, Alexandra 9320, New Zealand; c School of Science, The University of Waikato, Hamilton 3240, New Zealand; d Bay of Plenty Regional Council, 1 Elizabeth Street, Tauranga 3110, New Zealand

**Keywords:** Lakes, Chlorophyll, Remote sensing, Water quality, Monitoring, Spatial variability, C2RCC, Sentinel-2, MSI

## Abstract

Horizontal patchiness of water quality attributes in lakes substantially influences the ability to accurately determine an average condition of a lake from traditional in situ sampling. Monitoring programmes for lake water quality often rely on water samples from one or few locations but the assumption of representativeness is seldomly tested. Satellite observations can support environmental monitoring by detecting horizontal variability of water quality attributes over entire lakes. This article is a co-submission with Lehmann et al. (2021), who present a method to create a regional calibration of a satellite chlorophyll *a* algorithm and a spatial analysis of an image time series to detect recurring patchiness. Our method was developed on 13 lakes in the central North Island of New Zealand and this publication makes available the data used in our analysis and the spatial fields of results. These data are immediately valuable for practitioners operating within the region of interest providing a five year archive of synoptic water quality data and spatial fields to help optimize in situ monitoring efforts. In addition, there is value to the wider scientific community as the study lakes are a useful ‘natural lab’ for the development of aquatic remote sensing methods due to the range of trophic conditions and water colour in a single satellite image scene. Together with decades of in situ water quality records, our data is therefore useful for the development and validation of widely applicable methods of water quality retrieval from satellite data.

## Specifications Table

**Table:** 

Subject	Environmental Science
Specific subject area	Aquatic remote sensing
Type of data	Table Image Image time series (netCDF) Shapefile
How the data were acquired	***In situ* data**: Most water samples for chlorophyll *a* (*Chl*) determination were collected monthly as an integrated sample over the depth of the epilimnion from set monitoring stations shown in Fig. 1. Some samples were obtained from just below the water surface at varying locations. All samples were collected from a small boat. **Satellite data**: Data from the Multispectral Instrument (MSI) on Sentinel-2 satellites were downloaded as Level 1 files from the Copernicus API hub (https://scihub.copernicus.eu/apihub/) and processed with C2RCC (v1.5) [2] on Calvalus [3]. C2RCC's native Chl product was used for match-up selection. Regionally optimized Chl values determined from Inherent Optical Properties (IOPs) were applied for all further analysis (realized in Python 3.6)
Data format	Raw Analysed Filtered
Description of data collection	***In situ* chlorophyll *a* determination**: *Chl* analysis was done using acetone extraction of material filtered onto 1 μm glass-fibre filters and subsequent fluorometric measurement [4]. • **Satellite data processing:** • All available satellite scenes were processed with C2RCC (v1.5) [2] and Idepix, both with default settings, on Calvalus [3]; • Satellite data were resampled to 60 m pixel resolution; • Match-ups between in situ and satellite samples were generated under the following conditions: ○ Both data acquisitions occurred within 24 h of each other; ○ The 3 × 3 pixel region around the sampling station had at least six valid values (i.e. no raised quality flag, see Table 2); and ○ The relative coefficient of variation of satellite-derived *Chl* in the 3 × 3 pixel region was < 20%.
Data source location	• Country: New Zealand • Region: Rotorua Te Arawa Lakes, Bay of Plenty (37.973°S 176.202°N – 38.322°S 176.629°E) • Locations of water sampling locations are provided in Fig. 1
Data accessibility	Repository name: Zenodo Record number: 5336365 Direct URL to data: https://doi.org/10.5281/zenodo.5336364
Related research article	M. K. Lehmann, E. M. Schütt, M. Hieronymi, J. Dare, H. Krasemann, Analysis of recurring patchiness in satellite-derived chlorophyll a to aid the selection of representative sites for lake water quality monitoring, Int. J. Appl. Earth Obs. Geoinf. 104 (2021) 102547, doi: 10.1016/j.jag.2021.102547.

## Value of the Data

• The georeferenced spatial data over five years in thirteen lakes is useful for monitoring purposes and studies of freshwater ecology of the region.
• In situ samples and satellite match-ups from twelve optically diverse lakes are useful for the development of generally applicable methods for satellite water quality retrieval, atmospheric correction and ecological applications.
• The data benefits the regional monitoring agency and stakeholders of the lakes by providing an archive of water quality data and facilitating improvement of ongoing monitoring efforts.
• The data benefits the international aquatic remote sensing community by presenting a ‘natural lab’ useful for the development and validation of algorithm and processing methods.

## 1 Data Description

Here, we publish the final data products of our case study and intermediate products that are of use for local environmental agencies, ecologists and for the international aquatic remote sensing community.

**rotorua_chl_fields_2015-2020.nc** is a time series of 283 *Chl* fields of 13 of the lakes (Table 1) derived from Sentinel-2 MSI images with a regionalised parametrization of the C2RCC algorithm at 60 m pixel resolution [2]. It also includes C2RCC and Idepix [6] masks as well as a shoreline-and-shallow-water-buffer for flexible quality flagging.

**Table 1:** Characteristics of the Rotorua Te Arawa lakes.

Lake	Max. depth^a^ (m)	Surface area a (km²)	Lake centre	
			Lat	Lon
Ōkareka	34	3.3	–38.1715	176.3608
Ōkaro	18	0.3	–38.2987	176.3945
Ōkataina	79	10.7	–38.1286	176.4118
Rerewhakaaitu	16	5.2	–38.2960	176.5017
Rotoehu	14	7.9	–38.0190	176.5300
Rotoiti	126	33.7	–38.0358	176.4172
Rotokakahi	440	4.4	–38.2170	176.3207
Rotokawau	33	0.5	–38.0725	176.3769
Rotomā	83	11.1	–38.0440	176.5846
Rotomāhana	125	9	–38.2650	176.4427
Rotorua	45	80.5	–38.0790	176.2718
Tarawera	88	41.2	–38.1995	176.4315
Tikitapu	28	1.4	–38.1955	176.3314

a Data from [5]

**rotorua_chl_spatial_variability.tif** is a GeoTIFF that illustrates the representativeness of each grid cell for the *Chl* distribution in each lake and thus indicates recurring spatial patterns. The file contains three bands. Each band shows the relative frequency (in %) which *Chl* concentration was found near the median, or upper or lower quartile, respectively. The intervals around the median and quartiles are 5% to either side.

**Fig. 1:**
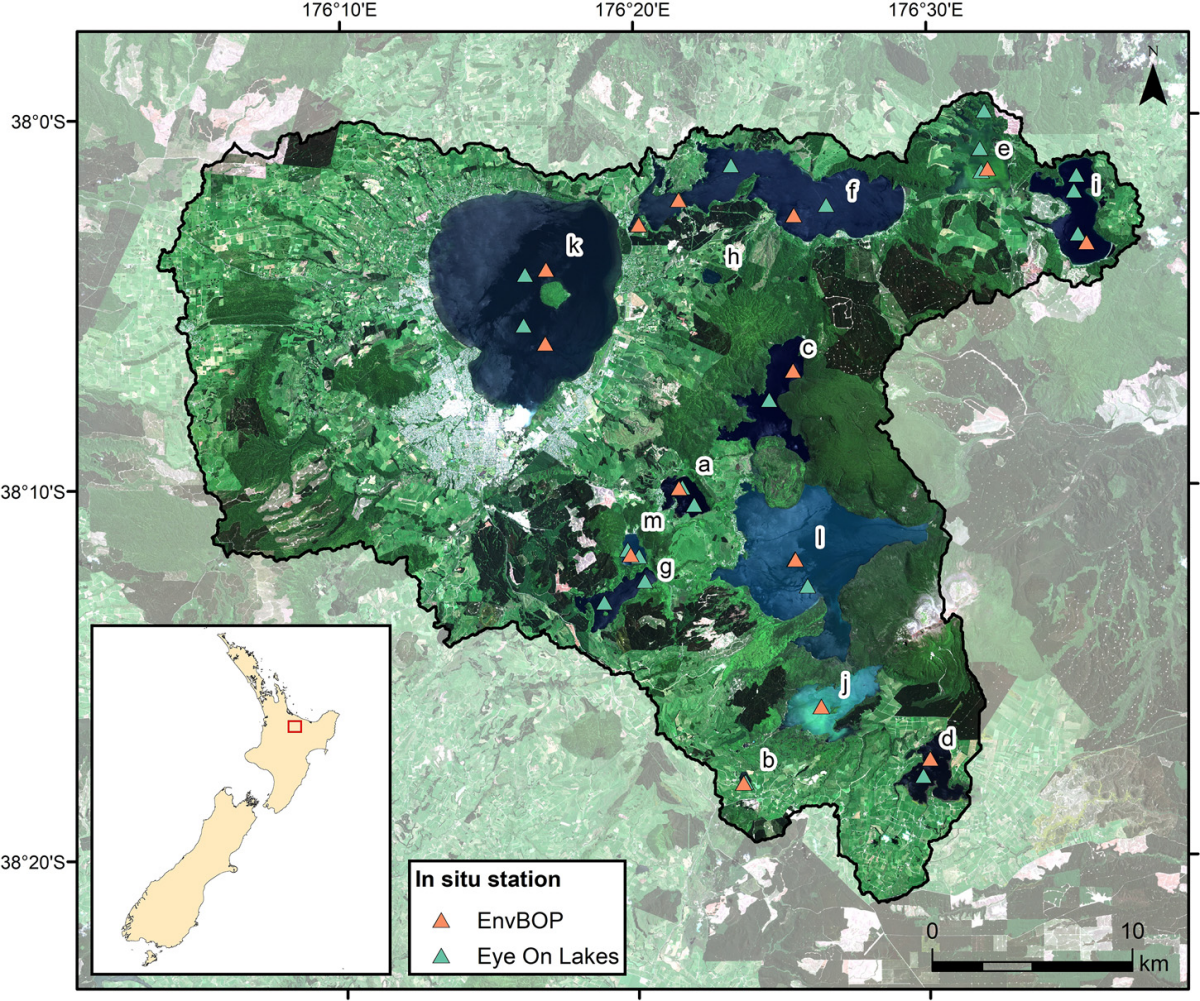
In situ sampling locations in the Rotorua Te Arawa Lakes. “EnvBOP” denotes to the Bay of Plenty lake water quality monitoring programme. The black line delineates the extent of the surface catchments for all lakes within the Rotorua Te Arawa Lakes complex. The background is a true color image of Sentinel-2B MSI scene taken on 17 December 2018 UTC. a: Ōkareka, b: Ōkaro, c: Ōkataina, d: Rerewhakaaitu, e: Rotoehu, f: Rotoiti, g: Rotokākahi, h: Rotokawau, i: Rotomā, j: Rotomāhana, k: Rotorua, l: Tarawera, m: Tikitapu.

**Fig. 2:**
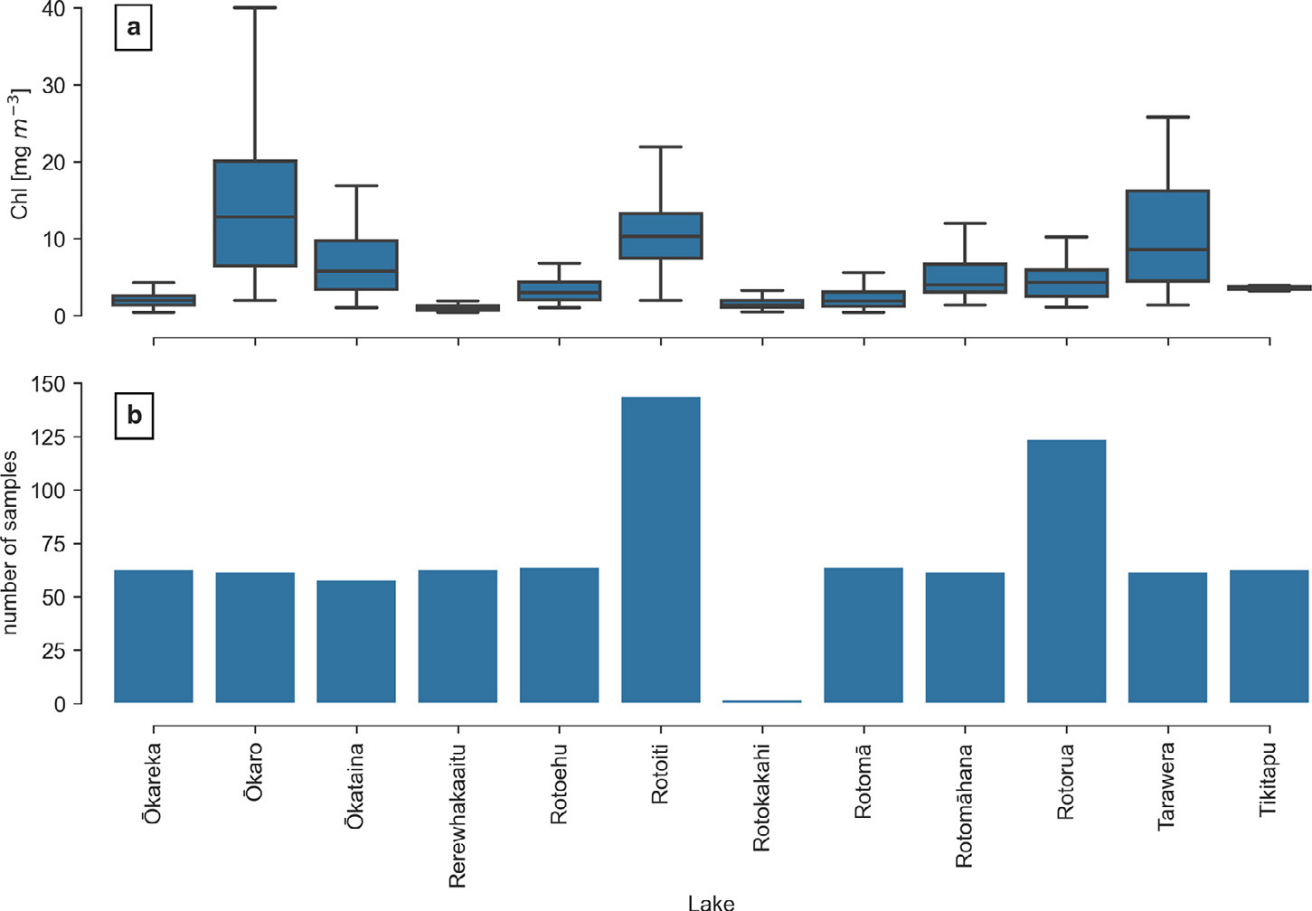
Range of measured *Chl* concentrations (a) and the total number of samples per lake (b).

**rotorua_insitu_chl_2015-2019.csv** contains 831 in situ *Chl* measurements from 12 of the lakes collected between 2015 and 2019. The majority of these measurements (802) have been taken as part of the monthly Bay of Plenty lake water quality monitoring programme, in which 11 lakes are monitored. The data set also contains samples from field work under the *Eye on Lakes* project (University of Waikato) obtained by one of the authors (MKL). These 29 samples also include two measurements at Lake Rotokakahi, which is not part of the monthly monitoring program. Fig. 1 shows all sampling locations and Fig. 2 the number of samples per lake and their summary statistics.

**shoreline_shallow_water_buffer.zip** contains a shapefile with polygons of the valid water pixels of all lakes to remove areas contaminated by bottom reflectance in remote sensing products. Each lake has a 120 m shoreline buffer to avoid mixed land-water pixels to reduce adjacency effects. It further excludes lake areas shallower than the 95%-quantile of all Secchi depth measurements of the Bay of Plenty lake water quality monitoring programme.

## 2 Experimental Design, Materials and Methods

### 2.1 *In situ* chlorophyll *a* sampling

The Bay of Plenty (BoP) Regional Council's lake water quality monitoring programme measures essential water quality parameters in 11 of the study lakes on a monthly basis [5]. Water samples and measurements are taken close to the deepest part of the lake as an integrated sample over the depth of the epilimnion (between 3 and 17 m). In lakes Rotorua and Rotoiti, two locations are sampled regularly (see Fig. 1 for their locations).

Between 2015 and 2019, 802 chlorophyll *a* (*Chl*) samples were taken within this monitoring programme. 29 additional samples were collected under the *Eye on Lakes* project led by one of the authors (MKL) at the University of Waikato. Some *Eye on Lakes* samples were obtained at locations different to those of the BoP stations. They also include two measurements from Lake Rotokakahi, which is not regularly sampled by BoP water quality monitoring programme. All locations are provided in the csv file (rotorua_insitu_*chl*_2015-2019.csv).

*Chl* and phaeopigments were determined fluorometrically according to APAH method 10300H [4]. Depending on the greenness of the water, a sample of 10 to 250 mL was filtered onto a 25-mm GF/C filter (Whatman). Filters were folded in half, wrapped in foil, and frozen. For analysis, samples were extracted in 90% acetone and *Chl* was determined by fluorometric assay (excitation at 430 nm and emission at 663 nm) before and after acidification.

### 2.2 Satellite data preparation

In this study, data from the Sentinel-2 Multispectral Instruments (MSI) were used. 282 satellite scenes of our study area between November 2015 and November 2020 were downloaded as Level-1C product from the Copernicus API Hub (https://scihub.copernicus.eu/apihub/) and the long-term archive into the *Calvalus* system [3]. All scenes were resampled to the resolution of the coarsest band (60 m) and the Case-2 Regional CoastColour algorithm (C2RCC version 1.5; [2]) was applied to remove atmospheric influences and retrieve inherent optical properties of the water and *Chl* concentrations. To identify and eliminate cloud contaminated pixels, all scenes have also been processed with the cloud screening and pixel characterisation tool IdePix [6] (see Table 2 for a description of all applied flags).

**Table 2:** Applied quality flags of C2RCC and IDEPIX and their descriptions. A pixel was valid if all flags with a “+” and no flags with a “–” were raised.

Flag	+/–	Description
IDEPIX_WATER	+	Water pixels
IDEPIX_CLOUD	–	Opaque clouds and semi-transparent clouds or clouds with uncertain detection level
IDEPIX_CLOUD_BUFFER	–	Buffer of 2 pixels around IDEPIX_CLOUD flag
IDEPIX_CLOUD_SHADDOW	–	Pixel is affected by cloud shadow
IDEPIX_CIRRUS_SURE	–	Cirrus clouds with full confidence of their detection
c2rcc_flags.Rtosa_OOS	–	Spectral shape of TOA input is not known to C2RCCs neural net
c2rcc_flags.Rtosa_OOR	–	Spectrum of TOA input is out of training range of C2RCCs neural net
c2rcc_flags.Rhow_OOR	–	One of the inputs to the IOP retrieval neural net is out of training range
c2rcc_flags.Rhow_OOS	–	Spectral shape of water leaving reflectance input spectrum to the IOP retrieval neural net is out of training range
c2rcc_flags_IOP_OOR	–	One of the IOPs is out of range
Shoreline and shallow water buffer	+	Contains areas further then 120 m to the shore and optically deep lake areas (not contaminated by bottom reflectance)

A special effort was made to mask areas near the shore and shallow water to remove potential mixed land-water pixels and areas where bottom reflectance may occur. A 120 m wide shoreline buffer (based on lake outlines in [7]) was created in QGIS (version 3.12). Shallow lake areas outside this buffer were identified based on bathymetric data provided by the BoP Regional Council and Secchi depth measurements of the BoP water monitoring programme. Lake areas shallower than the 95% quantile of all Secchi depth measurements of each lake were removed from the polygon. Finally, the “shoreline and shallow water”-mask was rasterized to the same grid as the satellite data, added to each scene and flagged in the further analysis.

### 2.3 Match-ups and optimized chlorophyll *a* product

C2RCC's native *Chl* product was compared to the in situ samples, if the time difference between the satellite overpass and the in situ measurement was within 24 hours. In this case, a macropixel (three-by-three pixels, i.e., 180 m × 180 m) centred at the sampling station was extracted with the match-up function in Calvalus. To ensure the comparability of an in situ point measurement and an area measurement of several squared meters of the satellite sensor [8], further tests were conducted in Python. A match-up was created, if at least six pixels of the macropixel were valid (i.e., not flagged; Table 2) and homogeneous. A macropixel was considered as homogeneous, if the standard deviation of all valid pixel values was less than 20% of its median [9]. If these criteria were passed, the match-up value was calculated as the mean of the valid macropixels *Chl* values.

The match-up samples were used to generate a regional calibration of the C2RCC *Chl* estimate by re-fitting the constituent absorptions of detritus ($a_{det}$) and CDOM ($a_{cdom}$) to the pigment absorption ($a_{pig}$):

(1) $$Chl=21{\left(a_{pig}+0.77\left(a_{det}+a_{cdom}\right)\right)}^{1.04}$$

This regionally optimized *Chl* product showed a high coefficient of determination (*r*² = 0.79) and acceptable errors (*RMSE* = 5.4 mg m³; see [1] for more information). The regionally optimized *Chl* product according to Eq. (1) for each of the 282 scenes and 13 of the lakes (Table 1) is provided in a single netCDF4-file (rotorua_chl_fields_2015-2020.nc).

### 2.4 Spatial summary statistics

**Fig. 3:**
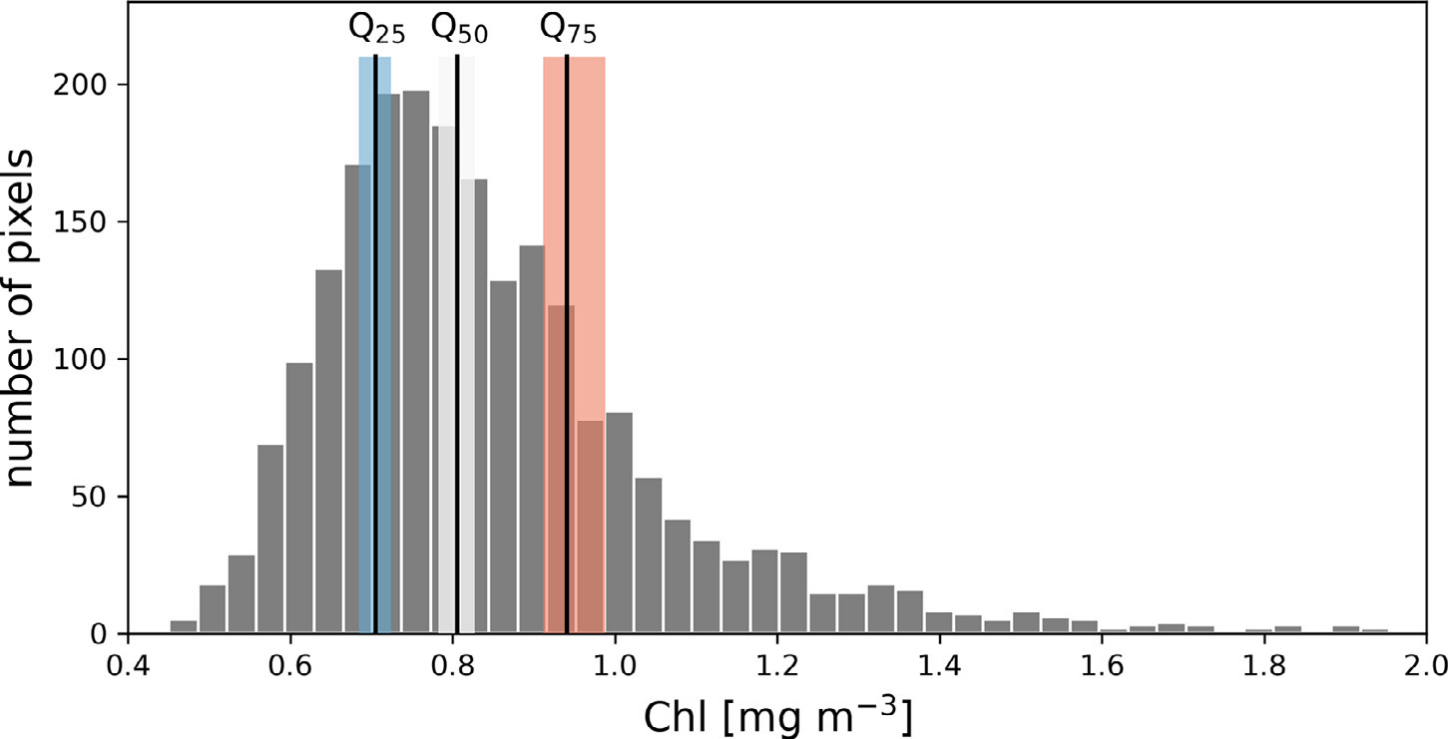
Histogram of *Chl* from a Sentinel-2 image of Lake Rotoehu taken on 12 December 2016 [1]. The positions of the median (Q_50_), lower (Q_25_) and upper (Q_75_) quantiles are shown as vertical lines. The shaded areas around each quantile indicate the ±5% range used for the category assignment.

Recurring spatial patterns of *Chl* were identified by analysing the frequency distributions of the *Chl* concentrations in each lake's satellite image with at least half of the lake's pixels not obscured by clouds. We identified pixels with *Chl* within 10 % of the lake's median, the lower quantile (25 % percentile), or the upper quantile (75 % percentile; see Fig. 3 for an example) and converted the number of assignments across all scenes to a relative frequency.

Lake areas where the *Chl* concentrations are often close to the median are particularly suited for regular water sampling, as measurements in this area maximise the representativeness of a point measurement for the whole lake. Additional sampling locations in areas where *Chl* is mostly close to the upper and lower quantile may further enhance the monitoring, as it provides a rough estimate on the spread of the lakes *Chl* distribution. Composite maps showing each pixel's quantile assignment are provided as a GeoTIFF (rotorua_chl_sp_var.tif).

## CRediT authorship contribution statement

**Eike M Schütt:** Methodology, Software, Writing – original draft, Visualization. **Moritz K Lehmann:** Conceptualization, Methodology, Data curation, Writing – review & editing. **Martin Hieronymi:** Methodology, Writing – review & editing. **James Dare:** Data curation, Writing – review & editing. **Hajo Krasemann:** Writing – review & editing. **Darryn Hitchcock:** Data curation. **Amy Platt:** Data curation. **Klay Amai:** Data curation. **Tasman McKelvey:** Data curation.

## Declaration of Competing Interest

The authors declare that they have no known competing financial interests or personal relationships that could have appeared to influence the work reported in this paper.
